# The Moderating Effects of Perceived Severity on the Generational Gap in Preventive Behaviors during the COVID-19 Pandemic in the U.S.

**DOI:** 10.3390/ijerph18042011

**Published:** 2021-02-19

**Authors:** Yunjuan Luo, Yang Cheng, Mingxiao Sui

**Affiliations:** 1 Department of Online Communication, School of Journalism and Communication, South China University of Technology, Guangzhou 510006, China; yunjuan.luo@yahoo.com; 2 Department of Communication, North Carolina State University, Raleigh, NC 27695, USA; 3 Department of Media & Communication, School of Arts and Science, Ferrum College, Ferrum, VA 24151, USA; msui@ferrum.edu

**Keywords:** risk perception, perceived severity, preventive behavior, generational gap, COVID-19

## Abstract

During the COVID-19 pandemic, older adults appear to be more susceptible to the coronavirus disease. Although the health stakes are higher for older adults, individuals of all ages should adopt preventive measures to contain the human-to-human transmission of the virus. This study conducted a national online survey of 1843 adults at the early stage of the COVID-19 pandemic in the U.S. to examine age disparities in preventive behaviors against the virus. The results show that older generations, motivated by a higher perceived severity of the disease, were more likely to take the precautionary actions recommended by the U.S. Centers for Disease Control and Prevention (CDC) than younger generations. This thus suggests that persuasive health messages addressing the severity of COVID-19 might reduce the generational gap and promote preventive behaviors among young people, to protect themselves and the elderly.

## 1. Introduction

### 1.1. Background

The coronavirus disease 2019 (COVID-19) pandemic has rebooted Americans’ debate over the effectiveness of mask-wearing that arose during the 1918–1919 influenza epidemic [1], which has also become part of the political terrain. Differently from many Asian nations, such as China and South Korea, that have enforced a universal masking policy since the initial stage, public health officials in the U.S. first denied the requirement of mask-wearing in February but then recommended the wearing of facial masks as a supplemental measure to hygiene and social distancing in early April 2020. This has led to widespread skepticism about the masking policy among Americans, regardless of extensive scientific findings that have shown that mask-wearing, along with the implementation of social distancing, is an effective and low-cost non-pharmaceutical measure to flatten the epidemic curve [2,3].

In particular, stark disparities have been uncovered between the young and elderly generations in terms of their willingness to wear a facial mask, and engagement in other preventive behaviors. For example, while 53% of people aged 60 and above self-reported to have engaged in all six Centers for Disease Control and Prevention (CDC)-recommended mitigation behaviors, including face covering, hygiene, social distancing, avoiding crowded places, cancelling or postponing social or recreational activities, and reducing restaurant dine-ins, only about 38% of young adults aged 18–29 years practiced all these methods [4]. In addition, although elderly people tend to wear masks in almost any settings, the young generation prefers not to wear facial masks when hanging out with their peers [5]. This may translate into about a 20% increase in the likelihood for those 65 years and older to wear masks than those who are under 30 years old [6]. Further nuances were yielded by Kim and Crimmins’s study [7], which found that at the beginning of the pandemic (March 2020) older people were actually not more likely to engage in preventive behaviors, including the practices of hygiene, face covering, quarantine, social distancing, etc., than the young generation; however, as the pandemic evolved (i.e., May 2020), older adults then exhibited a much greater compliance with preventive regulations.

Therefore, what could have contributed to this gap in preventive behaviors against the virus? One important factor might be the perceived severity of COVID-19, which was found to be positively associated with preventive behaviors in previous studies. For example, research shows that the more sever individuals perceive COVID-19 to be, the more likely they will stay at home during the pandemic [8]. However, as young adults tend to perceive a lower level of severity of virus outcome for themselves than for elderly populations [5], especially given the continuing disproportionate emphasis of the detrimental impact of COVID-19 on older people that can be found in the news media and other conversations [9], they may have fewer concerns about contracting COVID-19, and thus will be less likely to follow the CDC’s guidelines.

Yet, the line of research on COVID-19 so far has mostly focused on generational differences in prevention behaviors against the virus [6,7], without addressing the possible moderating role of perceived severity under this global health crisis. To fill in this gap in the current literature, this study seeks to explore age disparities in the perceived severity of COVID-19 and in the adoption of preventive measures, followed by applying extended statistical models to investigate how the perceived severity of the virus influences the generational gap in preventive behaviors.

### 1.2. Hypotheses

Risk perception refers to an individual’s subjective assessment of threats existing in the outside world that have a significant bearing on the individual’s subsequent responses [10]. The health belief model (HBM) identified two dimensions of the perceived threat of a health risk: perceived susceptibility, and perceived severity [11,12]. Perceived susceptibility refers to an individual’s perception of the chance of contracting a disease; whereas perceived severity refers to an individual’s opinion of how serious a disease and its consequences are.

Demographic characteristics are well-documented to affect people’s risk perception. Age-related differences in risk perception of a pandemic were found in past research. In the context of the avian influenza outbreak, elderly residents in Hong Kong reported a higher perceived susceptibility and a higher perceived clinical severity of outcomes [13]. In Australia, researchers found that older people reported having the highest level of threat perception, and feeling more susceptible to being affected by a possible influenza pandemic [14]. In a recent study assessing perceptions of risk concerning the COVID-19 epidemic among residents in Chongqing city, China, older people were found to have a higher level of risk perception towards infectivity, pathogenicity, lethality, and self-rated infection [15]. In this study on the COVID-19 pandemic in the U.S., we focused on the assessment of the public’s perceived severity of the coronavirus. Without preexisting immunity to this novel coronavirus, it is believed that everyone is susceptible to COVID-19; however, it has been often compared to the flu, and the danger of COVID-19 has been downplayed by some American media and politicians. Such misinformation could have an impact on the public’s belief in the threat of this disease [16]. Based on previous findings of age disparity in risk perception, we hypothesize that older age is associated with higher perceived severity of the disease.

**Hypothesis** **1** **(H1).** *Older generations would perceive COVID-19 to be severer than younger generations.*

Age, as a key demographic variable, not only affects public risk perceptions, but also associates with preventive behaviors. A meta-analysis study identifying demographic determinants of protective behaviors found that increase in age is largely associated with a greater chance of adopting preventive behaviors during the outbreak of pandemic influenza [17]. Cross-sectional studies of severe acute respiratory syndrome (SARS)-related behaviors in Singapore and Hong Kong have found that older people were more likely to carry out precautionary behaviors such as hand washing, respiratory hygiene, and mask wearing [18,19,20]. An internet survey conducted at the beginning of the H1N1 swine flu outbreak in Mexico found that compliance with recommended protective behaviors increased with age [21]. In the event of a human-to-human H5N1 outbreak in Hong Kong, older people were found to be more likely to report self-protective behaviors [22]. In a recent study on mask-wearing behaviors during the COVID-19 pandemic in the U.S., researchers also found that elderly adults (>65 years old) wear masks more frequently than middle-age (30–65 years old) and young people (2–30 years old), because older people are at a higher risk of severe illness from COVID-19 [6]. In line with these findings, we thus hypothesize a positive relationship between age and the adoption of preventive behaviors.

**Hypothesis** **2** **(H2).** *Older generations would be more likely to take preventive actions than younger generations.*

Understanding the link between risk perception and preventive behaviors can be derived from protection motivation theory (PMT) [23,24], which posits that the decision on adopting protective action recommendations is governed by two distinct cognitive processes: the formation of risk perceptions, and the application of protective behavior [25]. The formation of risk perceptions is a process of “threat appraisal” of perceived severity and perceived susceptibility. Those who perceive the threat of a health risk to be serious and feel they are personally vulnerable are more likely to take preventative actions.

Previous research has demonstrated a positive relationship between high-risk perceptions and the public’s active response to an epidemic. In the case of Zika prevention in Puerto Rico, pregnant women’s risk perception increased after they participated in a community intervention program, which also motivated them to engage in self-protective behaviors, such as mosquito repellent use and long-pant wearing [26]. In a study focusing on the first wave of the 2009 H1N1 pandemic in Hong Kong, despite the lack of consistency in their findings, researchers detected that the lower perceived severity was, the fewer hygiene measures were adopted [27]. A recent study of identifying factors influencing risk for COVID-19 exposure among young adults aged 18–23 years in Winnebago County, Wisconsin, found that their low perceived severity of disease outcome might partly explain why young adults did not adhere to the CDC’s guideline to wear a mask, when not in physical contact with loved ones at risk [5]. On basis of previous findings, we hypothesize that the perceived severity of COVID-19 can help narrow down the generational gap in preventive actions.

**Hypothesis** **3** **(H3).** *As the perceived severity of COVID-19 increases, the gap in preventive behaviors between the younger and older generations gets smaller.*

## 2. Materials and Methods

### 2.1. Data Collection and Respondent Profile

After getting approval from the Institute Review Board in an American southeastern university, we conducted a national online survey in April 2020, and sent an anonymous link to 1995 panel participants aged 18 and above through a professional survey company. A total of 1843 participants who completed the survey were included for analysis in this study.

As shown in Table 1, our respondents composed 43.3% male (*n* = 798) and 56.7% female (*n* = 1045). By generation, 777 (42.2%) respondents were baby boomers (55 years old and above), followed by 354 (19.2%) Gen X (40–54 years old), 521 (28.3%) Gen Y (25–39 years old), and 191 (10.4%) Gen Z (18–24 years old). Regarding ethnicity, 76.7% of the respondents identified themselves as Caucasian/White (*n* = 1413), with 9.5% as Black or African American (*n* = 175), 6.2% as Latino/Hispanic (*n* = 115), 4.9% as Asian/Pacific Islander (*n* = 91), 1.8% as other races (*n* = 33), and 0.9% as Native American/American Indian (*n* =16). In terms of education, 28.3% of participants had a bachelor’s degree (four-year college degree), 21.8% some college or no degree, 18.6% had a high school diploma or equivalent, 14.1% a master’s degree, 12.4% an associate or technical degree (two-year college degree), 2.6% a doctoral degree, and 2.2% less than a high school diploma. A total of 783 participants (42.5%) had an annual household income of $40,000 or under, followed by $40,001–$60,000 (*n* = 320; 17.4%), $100,001 and higher (*n* = 315; 17.1%), $60,001–$80,000 (*n* = 264; 14.3%), and $80,001–$100,000 (*n* = 161; 8.7%). Regarding political partisanship, 736 (39.9%) respondents identified themselves as democrats, 615 as republicans (33.4%), 447 as independents (24.3%), and 45 as others (2.4%).

### 2.2. Variables and Measurements

Variables of interest in this study can be split into three parts, including two outcome variables, one predicting factor, and a set of control variables.

Relative to single-item measurement, multi-item scales are plausibly more advantageous in terms of measurement reliability and predictive validity [28]. Thus, following previous research [29,30] that measured perceived severity as a multi-item scale, this study captured this variable using three items including “I believe that COVID-19 is a deadly disease”, “I believe that COVID-19 can bring severe health problems”, and “I believe that COVID-19 is a serious threat to my health”. Based on past research that composed an index of precautionary behaviors [30,31,32], *preventive actions* was captured by using six items measuring the degree to which participants were likely to clean hands, wear a face mask outside, limit outdoor activities, avoid attending mass gathering, keep social distance with others, and avoid contact with people who are sick, respectively. A similar approach was also used to measure *COVID information*, using multiple items displayed in Table 2. Note that all close-ended items were measured on a five-point Likert scale where 1 = strongly disagree (or least likely) and 5 = strongly agree (or most likely).

The interitem reliability was then evaluated using Cronbach alpha coefficients, which ranged from 0.76 to 0.83, and thus indicated good measurement consistency [33]. Accordingly, we combined multiple items that measured the same construct to compute an average score of all these items as the value for a given construct. For example, *perceiver severity* was eventually measured as the average of three items (M = 4.15, SD = 0.81). Thus, *perceived severity*, *preventive actions* (M = 4.31, SD = 0.67), and *COVID information* (M = 3.06, SD = 0.98) were all measured as a multi-item, continuous scale.

For demographic variables such as age and gender, we followed the academic tradition of using a single survey question, as summarized in Table 2.

## 3. Results

As both dependent variables were measured using multi-item, continuous scales (see Table 2 for details), linear regression (OLS) models were appropriate [34], and are commonly adopted in public health research [30,35]. Therefore, OLS models were performed to test all the hypotheses. In Model 1 and Model 2, each of the two outcome variables, *perceiver severity* and *preventive actions***,** was regressed against the independent variable *age groups*, which is a four-level categorical factor, while controlling for the other confounding variables specified in Table 2 above. In Model 3, an interaction term “*age* X *perceiver severity*” was also added to test whether the gaps in preventive actions between four age groups varied depending on the level of perceived severity. Table 3 displays the results of all models used for analyses.

### 3.1. Generational Gaps in Perceived Severity of COVID-19

We start with hypothesis 1 that assumed older generations would have a higher perceived severity of COVID-19 than younger generations. As displayed in Model 1 (see Table 3), the beta coefficients on Gen Y (*b* = 0.21, *p* = 0.002 < 0.01), Gen X (*b* = 0.33, *p* = 0.000 < 0.001), and baby boomers (*b* = 0.55, *p* = 0.000 < 0.001) were all positive and statistically significant. This indicates that relative to the omitted baseline group “Gen Z,” Gen X, Gen Y, and baby boomers would perceive COVID-19 to be significantly more severe.

**Figure 1:**
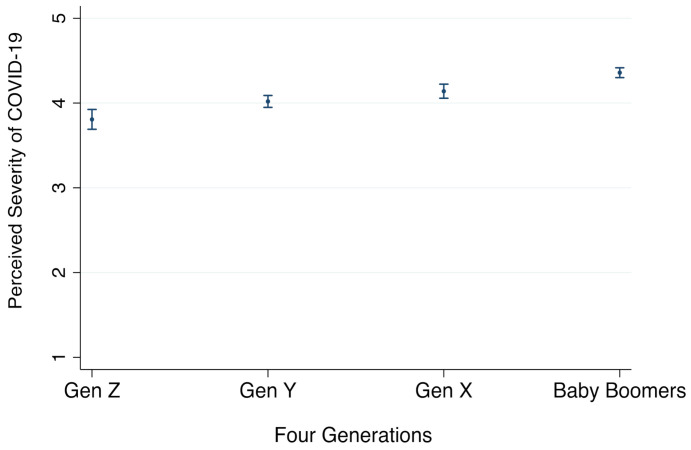
Comparison of Perceived Severity of COVID-19 by four generations, with 95% CI. Note: Perceived severity is measured as an average of three items, on a 5-point scale with bigger values indicating higher levels of perceived severity (see details in Table 1).

When comparing all four age groups to each other, the pairwise comparison results in Table 4 show that the difference in perceived severity between Gen X and Gen Y was not statistically significant. However, consistent with our expectations, baby boomers perceived COVID-19 to be significantly more severe than the younger generations, including Gen Z, Gen Y, and even Gen X. As such, among the four generations, Gen Z was the age group that tended to perceive the least severity of COVID-19, while baby boomers were the one that perceived it to be the most severe, as plotted in Figure 1. This thus yields support for H1.

### 3.2. Generational Gaps in Preventive Actions for COVID-19

**Figure 2:**
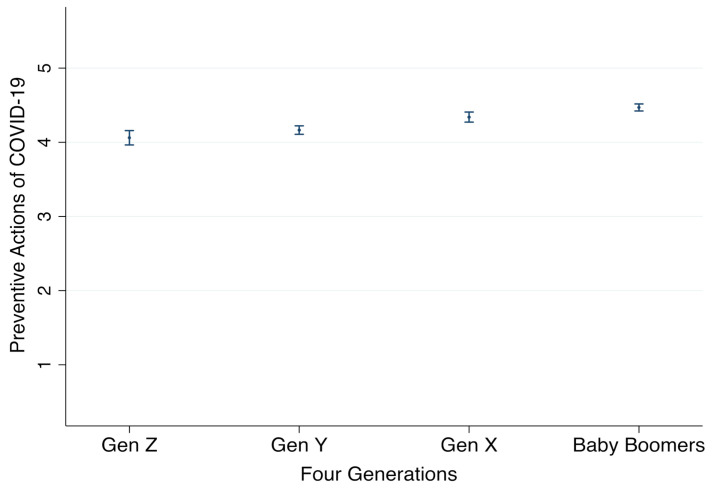
Comparison of Preventive Actions of COVID-19 by four generations, with 95% CI. Note: Preventive action is measured as an average of six items on a 5-point scale, with bigger values indicating that more preventive actions would be taken by respondents (see details in Table 1).

Now we move on to test hypothesis 2, which assumed that older generations would take more preventive actions than younger generations. As displayed in Model 2 (Table 3), relative to Gen Z, the other three generations, Gen Y (*b* = 0.10, *p* = 0.032 < 0.05), Gen X (*b* = 0.27, *p* = 0.000 < 0.001), and baby boomers (*b* = 0.41, *p* = 0.000 < 0.001), tended to take significantly more preventive actions. In addition, the results of pairwise comparisons also revealed that the differences between all generations were statistically significant, except for the contrast between Gen Y and Gen Z (difference = 0.10, *p* = 0.249). These results are plotted in Figure 2 for better interpretation; as illustrated, baby boomers tended to take significantly more preventive actions than the other three generations; similarly, Gen X would also use more preventive measure than Gen Y and Gen Z. Thus, H2 is supported.

### 3.3. Moderating Effect of Perceived Severity

Our third hypothesis investigated the moderating role of perceived severity, which assumed that the gap in preventive actions between the younger and older generations gets smaller, as the perceived severity of COVID-19 increases.

**Figure 3:**
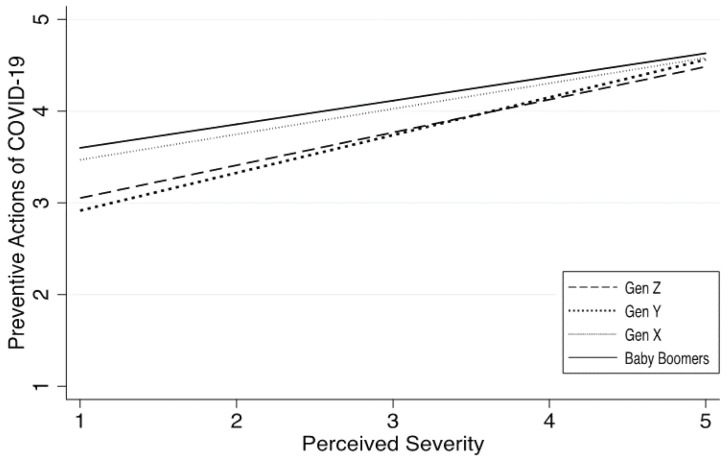
Comparison of preventive actions against COVID-19 by four generations, with 95% CI. Note: preventive action is measured as an average of six items on a 5-point scale, with bigger values indicating that more preventive actions would be taken by respondents (see details in Table 1).

This hypothesis was tested using the interaction terms between *Age* and *perceived severity* in Model 3, where only the coefficient on “*baby boomers X perceived severity*” was statistically significant (*b* = −0.10, *p* = 0.037 < 0.05), but not for the other two. Since the omitted reference age group was Gen Z, the negative coefficient indicates that the difference in preventive actions taken by Gen Z and baby boomers tended to decrease as the level of perceived severity increased. Figure 3 also plots these results for intuitive interpretations: when perceived severity was low (PS = 1), the predicted preventive actions made by Gen Z (dashed line) was 3.05, and 3.60 for baby boomers (solid line), with a difference of 0.44 on a 5-point scale; however, when the perceived severity increased to 5, the difference between Gen Z (M = 4.48) and baby boomers (M = 4.63) decreased to 0.15. Thus, H3 is partially supported.

Though not a focus of this study, Figure 3 also reveals an interesting pattern worthy of attention: The gap between baby boomers (solid line) and Gen X (dotted line) was minimal, indicating these two age groups behaved in very similar ways in terms of their preventive measures; on the other hand, Gen Y and Gen Z were adjacent to each other, and thus also seemed to share many similarities in their protective behaviors. This indicates that messages emphasizing the importance of preventive measures, such as wearing masks and social distancing, would be better targeted at a broader group, i.e., ages under 40, instead of concentrating on a smaller segment of young people under 29.

## 4. Discussion

As a highly contagious disease, COVID-19 spreads easily through person-to-person contact. Without good medical treatment and vaccines, preventive measures (e.g., mask wearing, social distancing, and avoiding large gatherings) are proven to be effective in controlling the rapid spread of the virus. Countries such as the United States that value individual rights and freedom are more reliant on the willingness of the general public to adhere to protective action recommendations than on containment or migration measures [22]. Given the link between risk perception and preventive behaviors documented in previous research [5,26,27], it is imperative to monitor public risk perceptions during public health emergencies, as part of emergence management to effectively control the transmission of infectious diseases.

Results from our study suggest that there is a generational gap in risk perception of COVID-19, and the perceived severity of the diseases increases with age. Compared to younger generations, baby boomers perceived the disease to be the most severe, given the fact that elderly people are at a higher risk of severe health consequences from COVID-19. The perceived severity of the virus was lower among the younger generations due to the mild nature of most cases.

Consistent with findings from previous research about the positive association between age and engagement in preventive behaviors [21,22,36], this study also found that older people tended to engage in more preventive actions than younger people. Those 18 to 24 years old were found to be the least likely to take protective actions. The lower percentage of younger generations adopting preventive measures, with their potential to be infected with no or mild symptoms [37], creates problems for infection control because they can keep the “chains of transmission” going, and put older adults and those with preexisting conditions of all ages at risk of severe illness [6].

Although this study focused on age differences in risk perception and preventive behaviors, the results also pointed to some other strong predictors (see Table 3). Democrats and those who received more COVID-related information perceived the disease to be more severe and reported more frequent engagement in precautionary measures. In line with findings from previous studies [18,19,36], gender was found to be another key factor associated with preventive behaviors. Compared to female participants, male participants were less likely to take precautionary actions.

The most notable finding of this study is that perceived severity can play a moderating effect in narrowing down the generational gap in preventive behaviors. As the level of perceived severity increases, the generational differences in the adoption of precautionary measures decreases. For younger age groups, especially Gen Y and Gen Z, who perceived low risk towards COVID-19, they might have underestimated the severity of this highly contagious disease, and were less likely to adopt self-protective behaviors. Therefore, they are the prime target for public health education. Persuasive health messages tailored for younger generations, to increase their level of perceived risk, may promote their preventive behaviors, which can help infection control among the target groups, and will be beneficial for the whole society.

Although young adults perceived a low level of severity of COVID-19 for themselves, past research also found that some of the youngsters reported their concerns about transmitting the virus to loved ones at risk and to the broader community [5]. Thus, communication messages designed to boost young adults’ social responsibility to protect others might also encourage them to adopt preventive measures to curb the spread of the virus in their communities.

## 5. Conclusions

The COVID-19 pandemic is a global health crisis. The U.S. continues to lead the world in new COVID-19 cases, with a total of more than 24 million confirmed cases and the death count surpassing 400,000 on 19 January 2020 [38]. Despite the controversy around face masking, the public’s compliance with protective measures plays a crucial role in controlling the spread of infectious disease.

Our study found age disparities in both risk perception and preventive behaviors during the early stage of the COVID-19 pandemic in the U.S. Compared to younger generations, older people perceived the disease as being more severe, and reported to be more likely to take CDC’s recommended protective actions. Perceived severity of the disease was found to be a key factor in reducing the generational gap in preventive behaviors. These results suggest that communication strategies and health education programs should focus on young people to raise their level of perceived severity of the pandemic disease, to encourage their compliance with protective measures.

Our study has a few limitations. First, our survey was cross-sectional in nature and was conducted in the early phase of the COVID-19 epidemic in the U.S. As the pandemic is continually getting worse, the public’s risk perceptions, attitudes, and behaviors may change with the evolution of the disease [14]. Given the fact that the cases of infection among young people are continually and rapidly increasing, the emphasis on disease severity in health communication messages to promote their precautionary actions remains relevant and critical for infection control. Second, although the public’s perceived severity of the virus was found to be a significant factor contributing to the increase in adopting protective behaviors, other important factors identified in theoretical health behaviors models such as response efficacy (the effectiveness of the recommended actions) and social norms need to be included for investigation in future research. Some realistic factors might also contribute to age disparities in preventive behaviors. For example, older people may be more aware of the limited access to health care amid the COVID-19 pandemic [39], so they tend to be more frequently engaged in protective behaviors than younger people. Third, the sample in the internet survey was not representative, and self-reported data might not accurately reflect the public’s attitudes and behaviors. Fourth, we adopted OLS models in the statistical analysis because the scale we used to measure dependent variables is coherent with statistical assumptions of the OLS method [34]. Some other statistical methods, such as logistical regression and structural equation modeling, might be appropriate for data analysis, which needs to be considered in future studies. Despite those limitations, our study offers useful evidence to inform public health officials and the media about the importance of addressing the severity of COVID-19 in health communication messages targeting young generations to help flatten the epidemic curve.

## Figures and Tables

**Table 1:** Respondent profile for the study (*n* = 1843).

Respondent Profile	*n*	*%*
Gender		
Male	798	43.3
Female	1045	56.7
Age		
18–24	191	10.4
25–39	521	28.3
40–54	354	19.2
55 and above	777	42.2
Race/Ethnicity		
Caucasian/White(non-Hispanic)	1413	76.7
Black/African American	175	9.5
Latino/Hispanic	115	6.2
Asian/Pacific Islander	91	4.9
Native American/American Indian	16	.9
Other	33	1.8
Annual Income		
$20,000 or under	392	21.3
$20,001 or 40,000	391	21.2
$40,001–$60,000	320	17.4
$60,001–$80,000	264	14.3
$80,001–$100,000	161	8.7
$100,001 and higher	315	17.1
Educational Level		
Less than higher school	41	2.2
High school diploma or equivalent	343	18.6
Some college but no degree	402	21.8
Associate or technical degree	228	12.4
Bachelor’s degree	521	28.3
Master’s degree	260	14.1
Doctoral degree	48	2.6
Political Partisanship		
Democrat	736	39.9
Republican	615	33.4
Independent	447	24.3
Other	45	2.4

**Table 2:** Summary of variables, survey questions, and measurements.

	Survey Questions	Measurement
Outcome Variables		
Perceived Severity (Cronbach α = 0.79)	I believe that COVID-19 is a deadly disease.	5-point scale where 1 = strongly disagree and 5 = strongly agree
	I believe that COVID-19 can bring severe health problems.	
	I believe that COVID-19 is a serious threat to my health.	
Preventive Actions (Cronbach α = 0.76)	Clean hands often.	5-point scale where 1 = least likely and 5 = most likely
	Wear a face mask outside.	
	Limit outdoor activities.	
	Avoid attending mass gathering.	
	Keep social distance with others.	
	Avoid close contact with people who are sick.	
Independent Variable		
Age	Please select your age from the following choices.	1 = Generation Z (18–24) 2 = Generation Y (25–39) 3 = Generation X (40–54) 4 = Baby Boomers (55 and above)
Control Variables		
Gender	What’s your gender?	1 = male and 0 = female
Partisanship	Generally speaking, do you usually think of yourself as a Republican, Democrat, independent, or what?	1 = Independent 2 = Republican 3 = Democrat
Ethnicity	Which of the following best describes your racial/ethnic identity?	1 = White 2 = Non-white (i.e., African Americans)
Education	What is the highest degree or level of education you have completed?	1 = less than high school diploma … and 7 = doctorate degree
Income	What is your annual income?	1 = $20,000 or under … and 6 = $100,001 and higher
Location	What is the state where you are living in?	1 = most affected states (NY, CA, FL, LA, IL, MA, MI, NJ, PA) 0 = less affected states (the other 41 states)
Personal Relevance	Is there anyone you know (e.g., family members, friends, colleagues, acquaintances) who have confirmed or suspected COVID-19?	1 = yes 0 = no
COVID information (Cronbach α = 0.83)	How much COVID-related information have you received from each of the following communication channels? (Broadcast television news, Cable television news, Print newspapers, Radio, Online News, Facebook, Twitter, Friends and family).	5-point scale where 1 = not at all and 5 = a great deal

**Table 3:** Linear Regression Models Predicting Perceive Severity and Preventive Actions.

	Perceive Severity	Preventive Actions	Preventive Actions
	Model 1 (H1)	Model 2 (H2)	Model 3 (H3)
Gen Y	0.21(0.002) **	0.10(0.032) *	−0.19(0.414)
Gen X	0.33(0.000) ***	0.28(0.000) ***	0.50(0.045) *
Baby Boomers	0.55(0.000) ***	0.41(0.000) ***	0.65(0.005) **
Perceived Severity (PS)	-	-	0.36(0.000) ***
PS X Gen Y	-	-	0.05(0.355)
PS X Gen X	-	-	−0.08(0.191)
PS X Baby Boomers	-	-	−0.10(0.037) *
Male	−0.06(0.135)	−0.16(0.000) ***	−0.14(0.000) ***
Republicans	−0.04(0.47)	−0.04(0.288)	−0.03(0.403)
Democrats	0.18(0.000) ***	0.09(0.026) *	0.03(0.373)
White	0.11(0.022) *	−0.02(0.575)	−0.06(0.106)
Educational Level	0.003(0.821)	−0.003(0.765)	−0.01(0.596)
Household Income	0.01(0.347)	0.01(0.238)	0.01(0.451)
Most Affected States	−0.01(0.853)	0.06(0.054)	0.06(0.025) *
Personal Relevance	0.03(0.468)	−0.05(0.20)	−0.05(0.107)
COVID information	0.19(0.000) ***	0.21(0.000) ***	0.15(0.000) ***
constant	3.05(0.000) ***	3.43(0.000) ***	2.32(0.000) ***
N of obs	1798	1798	1798
*R* ^2^	0.09 ***	0.12 ***	0.26 ***

Entries are beta coefficients with *p*-values in parentheses, where *** *p* < 0.001, ** *p* < 0.01, and * *p* < 0.05. The omitted baseline group is “Gen Z” for age groups, “Independents” for partisanship, and “non-whites” for ethnicity. See Table 1 for details of variable measurements.

**Table 4:** Pairwise Comparisons of Gen X, Y, Z, and Baby Boomers.

	Perceived Severity (Model 1)		Preventive Actions (Model 2)	
	diff.	*p*-Value	diff.	*p*-Value
Gen Z vs. Gen Y	−0.21	0.011	−0.10	0.249
Gen Z vs. Gen X	−0.33	0.000	−0.28	0.000
Gen Z vs. Baby Boomers	−0.55	0.000	−0.41	0.000
Gen Y vs. Baby Boomers	−0.34	0.000	−0.31	0.000
Gen X vs. Baby Boomers	−0.22	0.000	−0.13	0.013
Gen X vs. Gen Y	0.12	0.121	0.17	0.001

Entries are results with Tukey test for multiple comparisons.

## Data Availability

The data are not publicly available due to privacy or ethical restrictions.

## References

[1] Scerri M., Grech V. (2020). To wear or not to wear? Adherence to face mask use during the COVID-19 and Spanish influenza pandemics. Early Hum. Dev..

[2] Li T., Liu Y., Li M., Qian X., Dai S.Y. (2020). Mask or no mask for COVID-19: A public health and market study. PLoS ONE.

[3] Chu D.K., Akl E.A., Duda S., Solo K., Yaacoub S., Schünemann H.J. (2020). Physical distancing, face masks, and eye protection to prevent person-to-person transmission of SARS-CoV-2 and COVID-19: A systematic review and meta-analysis. Lancet.

[4] Hutchins H.J., Wolff B., Leeb R., Ko J.Y., Odom E., Willey J., Friedman A., Bitsko R.H. (2020). COVID-19 Mitigation behaviors by age group—United States, April–June 2020. Morb. Mortal. Wkly. Rep..

[5] Wilson R.F., Sharma A.J., Schluechtermann S., Currie D.W., Mangan J., Kaplan B., Euhardy N. (2020). Factors influencing risk for COVID-19 exposure among young adults aged 18–23 Years—Winnebago County, Wisconsin, March–July 2020. Morb. Mortal. Wkly. Rep..

[6] Haischer M.H., Beilfuss R., Hart M.R., Opielinski L., Wrucke D., Zirgaitis G., Uhrich T.D., Hunter S.K. (2020). Who is wearing a mask? Gender-, age-, and location-related differences during the COVID-19 pandemic. PLoS ONE.

[7] Kim J.K., Crimmins E.M. (2020). How does age affect personal and social reactions to COVID-19: Results from the national understanding America study. PLoS ONE.

[8] Irigoyen-Camacho M.E., Velazquez-Alva M.C., Zepeda-Zepeda M.A., Cabrer-Rosales M.F., Lazarevich I., Castaño-Seiquer A. (2020). Effect of income level and perception of susceptibility and severity of COVID-19 on stay-at-home preventive behavior in a group of older adults in Mexico City. Int. J. Environ. Res. Public Health.

[9] Petretto D.R., Pili R. (2020). Ageing and COVID-19: What is the role for elderly people?. Geriatrics.

[10] Solvic P. (1987). Perception of risk. Science.

[11] Becker M.H. (1974). The health belief model and personal health behavior. Health Educ. Monogr..

[12] Janz N.K., Becker M.H. (1984). The health belief model: A decade later. Health Educ. Q..

[13] Lau J.T.F., Kim J.H., Tsui H.Y., Griffiths S. (2007). Anticipated and current preventive behaviors in response to an anticipated human-to-human H5N1 epidemic in the Hong Kong Chinese general population. BMC Infect. Dis..

[14] Barr M., Raphael B., Taylor M., Stevens G., Jorm L., Giffin M., Lujic S. (2008). Pandemic influenza in Australia: Using telephone surveys to measure perceptions of threat and willingness to comply. Infect. Dis..

[15] He S., Chen S., Kong L., Liu W. (2020). Analysis of risk perceptions and related factors concerning COVID-19 epidemic in Chongqing, China. J. Community Health.

[16] Cheng Y., Luo Y. (2020). The presumed influence of digital misinformation in the U.S.: Examining publics’ support for governmental restrictions versus corrective action in the COVID-19 pandemic. Online Inform. Rev..

[17] Bish A., Michie S. (2010). Demographic and attitudinal determinants of protective behaviours during a pandemic: A review. Br. J. Health Psychol..

[18] Lau J.T.F., Yang X., Tsui H.Y., Kim J.H. (2003). Monitoring community responses to the SARS epidemic in Hong Kong: From day 10 to day 62. J. Epidemiol. Community Health.

[19] Leung G.M., Lam T.H., Ho L.M., Ho S.Y., Chan B.H.Y., Wong I.O.L., Hedley A.J. (2003). The impact of community psychological responses on outbreak control for severe acute respiratory syndrome in Hong Kong. J. Epidemiol. Community Health.

[20] Quah S.R., Hin-Peng L. (2004). Crisis prevention and management during SARS outbreak, Singapore. Emerg. Infect. Dis..

[21] Jones J.H., Salathe M. (2009). Early assessment of anxiety and behavioral response to novel swine-origin influenza A(H1N1). PLoS ONE.

[22] Lau J.T.F., Griffiths S., Choi K., Lin C. (2010). Prevalence of preventive behaviors and associated factors during early phase of the H1N1 influenza epidemic. Am. J. Infect. Control.

[23] Rogers R.W. (1975). A protection motivation theory of fear appeals and attitude change. J. Psychol..

[24] Rogers R.W., Cacioppo J., Petty R. (1983). Cognitive and physiological process in fear appeals and attitude change: A revised theory of protection motivation. Social Psychophysiology.

[25] Jiang X., Elam G., Yuen C., Voeten H., de Zwart O., Veldhuijzen I., Brug J. (2009). The perceived threat of SARS and its impact on precautionary actions and adverse consequences: A qualitative study among Chinese communities in the United Kingdom and the Netherlands. Int. J. Behav. Med..

[26] Earle-Richardson G., Prue C., Turay K., Thomas D. (2018). Influences of community interventions on Zika prevention behaviors of pregnant women, Puerto Rico, July 2016–June 2017. Emerg. Infect. Dis..

[27] Cowling B.J., Ng D.M., Ip D.K.M., Liao Q., Lam W.W.T., Wu J.T., Lau J.T.F., Griffiths S.M., Fielding R. (2010). Community psychological and behavioral responses through the first wave of the 2009 influenza A(H1N1) pandemic in Hong Kong. J. Infect. Dis..

[28] Diamantopoulos A., Sarstedt M., Fuchs C., Wilczynski P., Kaiser S. (2012). Guidelines for choosing between multi-item and single-item scales for construct measurement: A predictive validity perspective. J. Acad. Mark. Sci..

[29] Prasetyo Y.T., Castillo A.M., Salonga L.J., Sia J.A., Seneta J.A. (2020). Factors affecting perceived effectiveness of COVID-19 prevention measures among Filipinos during enhanced community quarantine in Luzon, Philippines: Integrating protection motivation theory and extended theory of planned behavior. J. Infect. Dis..

[30] Fathian-Dastgerdi Z., Khoshgofar M., Tavakoli B., Jaleh M. (2021). Factors associated with preventive behaviors of COVID-19 among adolescents: Applying the health belief model. Res. Soc. Adm. Pharm..

[31] Clark C., Davila A., Regis M., Kraus S. (2020). Predictors of COVID-19 voluntary compliance behaviors: An international investigation. Glob. Transit..

[32] Lin T.T.C., Bautista J.R. (2016). Predicting intention to take protective measures during haze: The roles of efficacy, threat, media trust, and affective attitude. J. Health Commun..

[33] Wang M.Y., Zhang P.Z., Zhou C.Y., Lai N.Y. (2019). Effect of emotion, expectation, and privacy on purchase intention in WeChat health product consumption: The mediating role of trust. Int. J. Environ. Res. Public Health.

[34] Hutcheson G.D., Moutinho L., Hutcheson G.D. (2011). Ordinary least-squares regression. The SAGE Dictionary of Quantitative Management Research.

[35] Sun Z., Yang B., Zhang R., Cheng X. (2020). Influencing factors of understanding COVID-19 risks and coping behaviors among the elderly population. Int. J. Environ. Res. Public Health.

[36] Li S., Feng B., Liao W., Pan W. (2020). Internet use, risk awareness, and demographic characteristics associated with engagement in preventive behaviors and testing: Cross-sectional survey on COVID-19 in the United States. J. Med. Internet Res..

[37] Davies N.G., Klepac P., Liu Y., Prem K., Jit M., Eggo R.M., CMMID COVID-19 Working Group (2020). Age-dependent effects in the transmission and control of COVID-19 epidemics. Nat. Med..

[38] McEvoy J.U.S. Hits 400,000 Covid-19 Deaths, Nearly 1 in Every 800 Americans. https://www.forbes.com/sites/jemimamcevoy/2021/01/19/us-hits-400000-covid-19-deaths-nearly-1-in-every-800-americans/?sh=7f3a337877c9.

[39] Szmuda T., Ali S., Sloniewski P. (2020). Telemedicine in neurosurgery during the novel coronavirus (COVID-19) pandemic. Pol. J. Neurol. Neurosurg..

